# Mecanismos de pago y gestión de recursos financieros para la consolidación del Sistema de Salud de Ecuador

**DOI:** 10.26633/RPSP.2017.51

**Published:** 2017-05-15

**Authors:** Tatiana Villacrés, Ana Cristina Mena

**Affiliations:** 1 Pontificia Universidad Católica del Ecuador Pontificia Universidad Católica del Ecuador Quito Ecuador Pontificia Universidad Católica del Ecuador,Quito,Ecuador.; 2 Ministerio de Salud Pública de Ecuador Ministerio de Salud Pública de Ecuador Quito Ecuador Ministerio de Salud Pública de Ecuador, Quito, Ecuador.

**Keywords:** Reforma sanitaria, financiamiento público, capitación, cobertura de los servicios de salud, Ecuador, Health care reform, financing, government, capitation fee, health services coverage, Ecuador

## Abstract

**Objetivos.:**

El objetivo de este artículo es analizar la propuesta planteada por el Ministerio de Salud Pública para la reforma del modelo de financiamiento público en Ecuador referente a mancomunación de fondos y mecanismos de pago.

**Método.:**

Se realizó una revisión documental sobre el modelo de financiamiento, el marco legal vigente y las bases presupuestarias por medio de Pubmed, Scielo, LILACS Ecuador y LILACS regional utilizando como palabras clave financiamiento de la salud, sistemas de financiamiento en salud, capitación, mancomunación de fondos, reforma de salud Ecuador, sistema de salud Ecuador y mecanismos de pago en salud. Se incluyeron, además, libros y otros documentos referidos por expertos en sistemas de salud.

**Resultados.:**

La revisión del modelo de financiamiento permitió identificar la segmentación histórica del sistema de salud ecuatoriano, a partir de la cual nace la propuesta del Ministerio de Salud Pública para reformar el modelo de financiamiento. El Ministerio ha planteado como soluciones la mancomunación de fondos y el pago de servicios en el primer nivel de atención mediante una cápita ajustada por riesgos socioeconómicos y demográficos. Los avances en la reforma del modelo de financiamiento incluyen el diseño de los planteamientos, sus mecanismos de implementación y el debate con los actores.

**Conclusiones.:**

La implementación de estas modificaciones puede generar mejoras para el sistema de salud en la eficiencia, dispersión de riesgos, incentivos para el cumplimiento de objetivos sanitarios, así como contribuir a su sostenibilidad y avanzar hacia la cobertura universal de salud. No obstante, existen limitaciones legales, políticas y operativas que dificultan su implantación.

## Abstract (en)

***Objective*.:**

Analyze the proposal by the Ministry of Public Health to reform the public financing model in Ecuador with regard to pooling of funds and payment mechanisms.

***Methods*.:**

A literature review was done of the financing model, the current legal framework, and the budgetary bases in Pubmed, SciELO, LILACS Ecuador, and regional LILACS using the key words health financing, health financing systems, capitation,pooling of funds, health system reform Ecuador, health system Ecuador, and health payment mechanisms. Books and other documents suggested by health systems experts were also included.

***Results*.:**

Review of the financing model enabled identifying the historical segmentation of Ecuador’s health system; out of this, the Ministry of Public Health conceived its proposal to reform the financing model. The Ministry’s proposed solutions are pooling of funds and payment of services at the first level of care through payment percapita adjusted for socioeconomic and demographic risks. Progress made in reforming the financing model includes design of the proposals and their implementation mechanisms, and discussions with stakeholders.

***Conclusions*.:**

Implementation of these changes may produce improvements for the health system in efficiency, spreading of risks, incentives for meeting health objectives, as well as contribute to its sustainability and advance toward universal healthcoverage. Nevertheless, legal, political, and operational constraints are hampering their implementation.

El acceso y la cobertura universales a la atención de la salud se encuentran actualmente en la agenda de los responsables de la toma de decisiones de la Región de las Américas y del mundo. En 2014, en una sesión del Comité Regional de la Organización Mundial de la Salud para las Américas, se aprobaron cuatro líneas estratégicas para fortalecer los sistemas de salud y avanzar hacia el acceso y la cobertura universales (1). El aumento y la mejora del financiamiento con criterios de equidad y eficiencia es una de las estrategias planteadas cuya importancia radica en que el financiamiento es una de las funciones de los sistemas de salud (2, 3).

En las diferentes regiones del mundo, el financiamiento de la salud se establece, entre otros elementos, en función de variables macroeconómicas,demográficas y fiscales, para decidir qué, cómo y a quién financiar. El financiamiento de la salud posee tres funciones: la recaudación de ingresos, la acumulación y gestión de recursos financieros, y la compra de servicios sanitarios (2, 4, 5).

El sistema público de salud de Ecuador se ha caracterizado históricamente por tener un financiamiento segmentado en el cual confluyen distintas fuentes de financiamiento y mecanismos de gestión de recursos financieros,asignaciones dispares y diferencias en las prestaciones y coberturas de los beneficiarios de cada subsistema público (4). Estas características son el resultado de una conformación histórica de los sistemas de salud en América Latina y el Caribe basada en la construcción jerárquica la sociedad (5).

La gestión actual del financiamiento público de los servicios de salud no se adapta a las necesidades que plantea la reforma del sistema. El fortalecimiento de la rectoría de la autoridad sanitaria, la dotación y formación del talento humano, las altas inversiones en infraestructura y equipamiento, la mejora de la calidad de la prestación de servicios, y los demás elementos de la transformación requieren disponer de los recursos suficientes y una gestión eficiente de los mismos para contribuir a la sostenibilidad del sistema. La dispersión de riesgos y los mecanismos ineficientes de asignación de recursos limitan el avance hacia la cobertura universal en salud (6).

A partir de la situación histórica del financiamiento público de la salud, se planteó desde el Ministerio de Salud Pública (MSP), entendido como la Autoridad Sanitaria Nacional (7), una reforma que contempla la mancomunación de fondos como mecanismo para corregir la segmentación del financiamiento del sistema público de salud. La mancomunación se define como la agrupación de recursos financieros prepagados y su gestión con el fin de dispersar (redistribuir más homogéneamente entre la población sana y enferma y no concentrar) riesgos para la salud de la población en el uso de los servicios de salud (2, 6). Este mecanismo es utilizado mayoritariamente por los países que avanzan hacia la cobertura universal (6).

La mejora de la eficiencia en la asignación de recursos incluye el pago per cápita ajustado por riesgo en el primer nivel de atención en sustitución de la asignación por presupuesto histórico. El pago per cápita ajustado por riesgos se define como un mecanismo mixto, prospectivo y retrospectivo, con riesgos compartidos entre el pagador y el prestador (8). La Constitución del Ecuador contempla que la distribución de los recursos económicos públicos se base en criterios poblacionales y de necesidades de salud (9), un mandato al que se ajusta la reforma propuesta del financiamiento. Además, este proceso exige mantener equilibrios entre los ejes de cobertura, cartera de servicios y proporción de gastos cubiertos, porque influyen decisivamente en el volumen de recursos y gasto y, por tanto, en el avance hacia la cobertura universal (10).

El objetivo de este artículo es analizar la propuesta planteada por el Ministerio de Salud Pública para la reforma al modelo de financiamiento público en Ecuador referente a mancomunación de fondos y mecanismos de pago.

## MATERIALES Y MÉTODOS

Se realizó una revisión de documentos sobre el modelo actual de financiamiento del Ecuador, su marco legal vigente y las bases de datos presupuestarias de cada uno de los subsistemas públicos de salud. Por otro lado, se recopiló la información ofrecida por el MSP sobre los fundamentos y elementos técnicos de la reforma del financiamiento público relativos a la mancomunación de fondos y al pago de una cápita en el primer nivel de atención.

Para disponer de sustento bibliográfico, se realizó una búsqueda de evidencia científica en Pubmed, Scielo, LILACS Ecuador y LILACS regional, en la cual no se consideró el año de publicación. Se utilizaron como palabras clave de búsqueda financiamiento de la salud, sistemas de financiamiento en salud, capitación, mancomunación de fondos, reforma de salud Ecuador, sistema de salud Ecuador, y mecanismos de pago en salud. Es importante señalar que también se efectuó una búsqueda con estas palabras en inglés, y que en Scielo también se encontraron documentos en Portugués. Los resultados de la búsqueda inicial de documentos no cribados se presentan en el cuadro 1 por fuente bibliográfica e idioma.

Con la documentación encontrada, se realizó una revisión de los títulos y resúmenes de los cuales sólo se seleccionaron los que hacían referencia a América Latina, Organización Mundial de la Salud (OMS), Organización Panamericana de la Salud (OPS) y a experiencias de implementación de reforma del financiamiento, con el afán de que la bibliografía obtenida pudiera aportar información al marco conceptual de este artículo.

Además de los artículos que se encontraron en esta búsqueda, se incluyeron libros y otros documentos referidos por expertos en sistemas de salud. Esto arrojó en total las 37 referencias utilizadas a la postre en este artículo.

## RESULTADOS

En los documentos encontrados se identificaron los principales elementos de la situación del financiamiento público de la salud en Ecuador, los problemas derivados del sistema actual, y las propuestas y avances en la reforma del modelo de financiamiento ecuatoriano.

### Financiamiento de la salud en Ecuador

En el caso ecuatoriano, en 2008 entró en vigencia la actual Constitución del Ecuador, que establece que la salud es un derecho que está garantizado por el Estado (9). Para cumplir este mandato, la Carta Magna establece que el Sistema Nacional de Salud, constituido por “instituciones, programas, políticas, recursos, acciones y actores en salud”, tanto públicos como privados, velará por el cumplimiento del derecho (7, 9). En cuanto al financiamiento indica que “será oportuno, regular y suficiente, y deberá provenir de fuentes permanentes del Presupuesto General del Estado. Los recursos públicos serán distribuidos con base en criterios de población y en las necesidades de salud…”(9). (Según el Código Orgánico de Planificación y Finanzas Públicas, los ingresos permanentes son aquellos que se reciben “de manera continua, periódica y previsible”. Este concepto se maneja para todo el Presupuesto General del Estado, independientemente del sector que reciba estos recursos.)

**CUADRO 1.** Resultados iniciales no cribados de la búsqueda bibliográfica

Dimensiones	Pubmed	SciELO	LILACS Ecuador	LILACS regional
Financiamiento de la salud	30 469	272	33	3 302
Sistemas de financiamiento de salud	4 799	0	6	2 666
Capitación	201	0	0	0
Mancomunación de fondos	8	0	0	162
Reforma de salud Ecuador	11	699	130	68
Sistema de salud Ecuador	15	0	133	183
Mecanismo de pago en salud	52	12	0	0

***Nota:*** Las cifras de los documentos incluyen los encontrados en todos los idiomas.

***Fuentes:*** Pubmed, Scielo, LILACS Ecuador, LILACS regional.

En el ámbito público del Sistema Nacional de Salud, forman parte de la Red Pública Integral de Salud (RPIS) el MSP, el Instituto Ecuatoriano de Seguridad Social (IESS), el Ministerio del Interior, el Instituto de Seguridad Social de la Policía Nacional (ISSPOL), el Ministerio de Defensa, y el Instituto de Seguridad Social de las Fuerzas Armadas (ISSFA) (11). El MSP actúa en el sistema como Autoridad Sanitaria (12), regulador y proveedor de servicios de salud, cuya fuente de financiamiento son los recursos del Presupuesto General del Estado. Dichos recursos son recaudados por el Ministerio de Finanzas y enviados al MSP, institución que los gestiona y compra bienes y servicios de salud (13). Lo mismo ocurre en los Ministerios del Interior y Defensa, que financian parcialmente las unidades de salud que se encuentran bajo su cargo. Por otra parte, las fuentes de financiamiento del seguro de salud del IESS son las aportaciones personales y patronales de los afiliados, que son administradas por la Dirección del Seguro General de Salud Individual y Familiar (14). El IESS, además de administrar sus recursos, es proveedor de servicios de salud al ser poseedor de varias unidades en los tres niveles de atención a escala nacional. A diferencia del IESS, el ISSFA y el ISSPOL son entidades exclusivamente aseguradoras de la policía nacional y las fuerzas armadas, respectivamente, cuyos seguros de salud provienen de las contribuciones patronales y personales (15).

**CUADRO 2.** Presupuesto devengado de las instituciones de la Red Pública Integral de Salud de Ecuador en miles de dólares corrientes, 2012-2015

Año								
Instituciones	2012 6,5		2013 7,3		2014 9,2		2015 ND	
Gasto total en salud (% PIB)	Presupuesto Devengado	Participación (%)	Presupuesto Devengado	Participación (%)	Presupuesto Devengado	Participación (%)	Presupuesto Devengado	Participación (%)
MSP	1 677,41	49,37	1 999,44	53,69	2 162,06	51,58	2 323,12	58,50
IESS	1 577,22	46,42	1 561,99	41,94	1 855,94	44,28	1 466,11	36,92
ISSFA	49,68	1,46	43,71	1,17	52,42	1,25	62,07	1,56
Fuerzas Armadas	55,15	1,62	63,40	1,70	69,97	1,67	71,34	1,80
ISSPOL Y Policía Nacional	38,34	1,13	55,85	1,50	50,94	1,22	48,77	1,23
Total	3 397,80	100,00	3 724,39	100,00	4 191,33	100,00	3 971,41	100,00

***Fuente:*** Ministerio de Finanzas, IESS, ISSFA, ISSPOL.

Como se indica en el cuadro 2, los subsistemas de las fuerzas armadas y la policía nacional son los miembros de la RPIS con menor cobertura y su gasto representa 4,6% del total de recursos devengados por la RPIS (16–18). El IESS dispuso de un presupuesto anual medio de 1 615,31 millones de $ US entre 2012 y 2015 con una participación de 36,9% (19). El MSP es el mayor financiador de sistema público con un gasto anual de 58,5% del gasto público (20).

#### Problemas derivados del sistema actual

Desde el ámbito de la función del financiamiento, el sistema ecuatoriano es segmentado, es decir, existen varias instituciones que ejercen las funciones de recaudación, mancomunación y compra de servicios. La segmentación tiene varias consecuencias, que se identifican a partir de las experiencias de distintos países, como la limitación de los subsidios cruzados entre los distintos grupos poblacionales, duplicidad de esfuerzos y gastos administrativos, falta de planificación integrada o menor posibilidad de financiar enfermedades con diagnósticos y tratamientos de alto costo (2, 4, 6, 20).

En la RPIS existen cuatro subsistemas, cada uno de ellos con sus propios recursos, mecanismos de distribución, gestión y evaluación. Como hay cuatro administradores de recursos, también existen diferentes equipos burocráticos y sistemas informáticos, así como distintas maneras de aplicar la normativa para la gestión y compra de prestaciones de salud. Esto ha complicado el acceso universal a los servicios de salud, puesto que existen diferencias en cuanto al alcance de la cobertura, los tiempos de espera y los pagos directos de la población ecuatoriana.

La formulación de los arreglos financieros en salud de un país afectan a los avances hacia un servicio de salud universal, ya que esto exige expandir la cobertura de servicios de salud y proteger financieramente a su población (21, 22).

La asignación de recursos y los mecanismos de pago del MSP se realizan con los presupuestos históricos en todos los niveles de atención. Este esquema corresponde a una integración del financiador y el proveedor en la compra de servicios. Dicho sistema de pago genera ineficiencias (6) por el incentivo a incrementar costos (23, 24), separa las transferencias monetarias de los resultados en salud, desincentiva el cumplimiento de objetivos sanitarios y la mejora de la calidad (25), y disminuye los incentivos para una provisión sanitaria eficiente (23, 26, 27). Asimismo, cuando la asignación no se ajusta a las necesidades de la población asignada a un establecimiento de salud, se puede propiciar inequidad en la distribución de recursos y, consecuentemente, en el acceso a los servicios.

El mecanismo actual tampoco ha facilitado cobros por parte de la red del MSP a los demás miembros de la RPIS por las prestaciones y servicios brindados en el primer nivel de atención a su población cubierta. Esto implica que, aunque no existe mancomunación de fondos, el financiamiento del primer nivel lo asume principalmente la Autoridad Sanitaria, al poseer la mayor red de establecimientos de este tipo (28). Por el contrario, en el nivel hospitalario existe un mecanismo de reconocimiento económico de las prestaciones de salud que brindan los miembros de la RPIS a la población cubierta por las demás instituciones públicas de salud (11).

#### Propuestas y avances en la reforma del modelo de financiamiento ecuatoriano

Para solventar el problema histórico de la segmentación, la OPS recomienda la mancomunación de recursos económicos, para dispersar de este modo los riesgos epidemiológicos y financieros entre la población sana y enferma (10). Aunque sean difícilmente extrapolables por los imperativos que suponen el contexto y las características específicas de cada país, las experiencias de diferentes países muestran que entre los potenciales impactos de un financiamiento mancomunado se encuentran el incremento del acceso a los servicios de salud; la reducción de las admisiones y readmisiones no planificadas de pacientes y de los costos totales de su atención; la mejora de los resultados en salud y la calidad; la disminución de la duración de las estancias; la sostenibilidad del sistema por dispersión de riesgos entre grupos poblacionales; la entrega del financiamiento en función de las necesidades de la población, y la compra de servicios usando las ventajas de las economías de escala (29–31).

En función de esta recomendación, la segmentación histórica del financiamiento en Ecuador, las necesidades del país y las experiencias en otros países (4), el MSP desarrolla una propuesta para estructurar el Fondo Nacional de Salud Ecuatoriano (FONSE). El FONSE se propone como una entidad adscrita al MSP encargada de la administración de los recursos públicos destinados al sector salud con excepción de los pertenecientes a la seguridad social (1 576,95 millones de $US en 2015) (30).

Se plantea que el FONSE tenga la función de recaudación, consolidación, gestión y evaluación de todos los recursos públicos para el pago de las prestaciones de salud. El FONSE sería el brazo ejecutor de las políticas de financiamiento elaboradas por el MSP como Autoridad Sanitaria. Sin embargo, la propuesta establece como una limitación, dada la imposibilidad Constitucional, el fusionar recursos del Presupuesto General del Estado conlos de la Seguridad Social, sin los cuales no existiría una verdadera mancomunación de recursos públicos para la prestación de servicios de salud. Se sugiere “redefinir constitucionalmente” la composición del Presupuesto General del Estado o diferenciar los recursos de la Seguridad Social de la siguiente forma:los recursos destinados al seguro de salud se transferirían al FONSE y los demás, continuarían cubriendo las otras prestaciones de aseguramiento social (30).

La formación de un fondo que mancomune los recursos de salud busca solucionar la segmentación del sistema público de salud ecuatoriano, que divide el alcance y el nivel de cobertura de la población y los costos administrativos. La creación del FONSE evitaría los múltiples equipos de auditoría médica, gestión y cobro, así como el planillaje (generación de planilla de reclamo de pago de atenciones brindadas) y la facturación en la RPIS. Por el contrario, esta instancia juntaría al equipo técnico y administrativo, ya existente y necesario para su operación, sin generar erogaciones administrativas adicionales, evitando así costos de transacción innecesarios y ordenando las funciones que actualmente se encuentran dispersas intra e interinstitucionalmente. Por otro lado, la mancomunación de fondos y riesgos en salud puede generar una solidaridad entre riesgos financieros, de edad y epidemiológicos, que ayude a la sostenibilidad de los sistemas de salud al generarse subsidios cruzados desde los que no necesitan los servicios de salud en la actualidad hacia los que los necesitan (4).

Por otro lado, para corregir el mecanismo histórico de asignación, el planteamiento desde la Autoridad Sanitaria contempla un sistema mixto prospectivo y retrospectivo que minimice el riesgo de selección de pacientes y maximice la eficiencia (23). Este diseño incluye la definición de un techo presupuestario para todos los niveles de atención basado en una cápita ajustada por factores socioeconómicos y demográficos por el lado de la demanda y de complejidad de la atención por el lado de la oferta. Los factores de ajuste considerados son la edad y el sexo de la población, el nivel de pobreza y la complejidad de los egresos hospitalarios. La incorporación de ajustes de riesgo permite incluir información sobre pacientes que motiven variaciones en el presupuesto y el uso de recursos de acuerdo con las necesidades de la población cubierta (31, 32).

El sistema de pagos que se plantea para el primer nivel de atención comprende una cápita a nivel de distritos ajustada por factores sociales, económicos y poblacionales. El monto para el primer nivel permite cubrir toda la cartera de servicios de promoción, prevención,curación y rehabilitación definida por tipo de establecimiento, lo que requiere la homologación de servicios en los establecimientos de salud (33). Es así como el riesgo se comparte entre prestadores y financiadores (24) y como se generan incentivos para que los proveedores reduzcan sus gastos y sean más eficientes (23, 34).

Una condición necesaria para la implementación de un pago capitativo es la adscripción territorial de la población a unidades de salud de primer nivel de la RPIS. La propuesta también incorpora un método para desarrollar este componente como un proceso de inscripción del usuario a un establecimiento para la utilización de sus servicios de salud durante un período específico y cumpliendo las responsabilidades definidas para el proceso (33), y considera, asimismo, la incorporación de incentivos financieros sobre la base de pagos por desempeño a las unidades de salud en el pago para el cumplimiento de objetivos de salud y la mejora de la eficiencia (6).

El pago mediante una cápita puede desincentivar la sobreutilización de los servicios (característica de los sistemas de pago por acto, *fee-for-service*), lo cual puede contribuir de este modo a mejorar la eficiencia en el gasto y motivar las acciones preventivas y el cuidado del paciente (6). Cuando la cápita se ajusta según las necesidades de la población, una parte del riesgo se traslada al prestador, y ello puede disminuir la selección de pacientes que generan menores costos (33) y mejorar la calidad del servicio (25). La distribución de recursos que propicia este mecanismo, si se diseña adecuadamente (31), permite, además, destinar mayores recursos a territorios con más necesidades, lo cual promueve una asignación de recursos con mayor equidad y el cumplimiento de objetivos en salud (25). Otra ventaja adicional de la capitación es la coordinación y articulación de niveles asistenciales (31, 35) dependiendo del contexto de la implementación (36).

Aunque, como en cualquier otro sistema de pagos, existen desventajas relacionadas con la motivación para derivar a los pacientes al segundo nivel de atención de manera anticipada, no brindar atención especializada o motivar la subutilización de servicios, los beneficios pueden ser mayores. Al igual que otros mecanismos de pago, el pago por cápita ajustado necesita ser controlado para asegurar que la selección, la disponibilidad limitada de recursos, la relación capacidad instalada/población adscrita y los posibles desajustes en las indicaciones y la utilización no deriven en restricciones al acceso de los servicios.

### DISCUSIÓN

Los cambios en el financiamiento son un elemento sustancial para todo el proceso de reforma del Sistema Nacional de Salud en Ecuador. La permanencia de esta transformación requiere una asignación adecuada de recursos financieros y su gestión eficiente para garantizar la sostenibilidad del sistema.

El modelo vigente de financiamiento público, con su construcción histórica, no contribuye a la conformación de un sistema de salud sostenible. Las distintas fuentes de financiamiento, gestión de los recursos financieros, coberturas diferenciadas y mecanismos de asignación de recursos no responden a las necesidades que plantean los cambios realizados en el sistema.

En este contexto, el MSP replantea el sistema segmentado, contemplando cambios en la mancomunación y asignación de recursos de acuerdo con las necesidades de la población en busca de la cobertura universal y la eficiencia. La experiencia de otros países en la implementación de estos esquemas, así como los potenciales resultados en optimización de recursos, eficiencia y mejora de la calidad de la prestación de servicios de salud, brindan elementos para sustentar la reforma del financiamiento planteada. Sin embargo, los resultados esperados están sujetos tanto a factores institucionales y del entorno, como a aspectos relacionados con las características de la implementación y la consolidación del proceso a lo largo del tiempo.

La creación del FONSE permite agrupar los recursos públicos que financian la cobertura de salud y dispersar los riesgos de la población. La Autoridad Sanitaria ha generado capacidades de gestión propias para avanzar hacia la reforma del financiamiento, creando cambios en la estructura orgánica del MSP donde se institucionalizaron áreas técnicas y estratégicas dedicadas a la generación de insumos técnicos respecto a financiamiento y costos, la articulación de la relación con las entidades de salud públicas y privadas, la generación de política de financiamiento, y la administración de convenios, entre otras necesarias para el funcionamiento del FONSE (37). Estos equipos técnicos han sido capacitados y han tenido un seguimiento continuo por parte de organismos nacionales e internacionales.

Actualmente, el MSP trabaja en la instauración de la carrera sanitaria del recurso humano operativo y estratégico para consolidar estas capacidades. Sin embargo, el ordenamiento del financiamiento disponible y su distribución necesitan, además de la voluntad política para delegar la administración de los recursos a una institución diferente, reformas del marco legal vigente que faciliten la reestructuración.

Un elemento importante de trabajo tanto para la reforma del financiamiento como para la consolidación de la RPIS es la adscripción territorial y la homologación de la cartera de servicios. También son relevantes la definición, caracterización y delimitación de la profundidad de la cobertura de servicios de salud teniendo en cuenta las dimensiones del cubo de Busse para establecer a quién, qué y cuánto se debe cubrir, esto a su vez considerando que la universalidad y gratuidad de los servicios públicos generan una amplia demanda que debe cubrirse con recursos limitados. Estos elementos son requisitos de obligatorio cumplimiento para avanzar en la transformación del modelo.

Las principales dificultades que se encontraron en el proceso de construcción de las propuestas de reforma se centraron en la disponibilidad de información desagregada respecto a costos de servicios de salud, población beneficiaria de cada subsistema y cartera de servicios. Pese a estas limitaciones, se logró diseñar una propuesta utilizando estimaciones a partir de los datos disponibles.

Sin embargo, aunque el planteamiento de reformar el financiamiento está diseñado, su implementación está pendiente. Para cumplir con este propósito, la participación activa y el consenso de los subsistemas públicos de salud se constituyen en un elemento clave. No obstante, se encontraron obstáculos para lograr acuerdos en la operativización de los mecanismos planteados, desde el ámbito legal hasta la reorganización interna de los subsistemas para adaptarse a los cambios.

Como cualquier otro proceso de cambio, la decisión política es el factor detonante de la ejecución de la reforma, sobre todo, cuando ello implica gestión de recursos. Tanto para la creación del FONSE, como para la incorporación de un pago capitado en el primer nivel de atención, existió voluntad política al máximo nivel. Sin embargo, fue a nivel jerárquico superior de los distintos sistemas de seguridad social donde se generaron puntos de desencuentro basados en la realidad de cada uno y en la posible reacción de la población que cubren.

#### Agradecimientos.

Los autores agradecen a Cecilia Acuña sus aportaciones a la elaboración de este artículo.

#### Declaración.

Las opiniones expresadas por los autores son de su exclusiva responsabilidad y no reflejan necesariamente los criterios ni la política de la Organización Panamericana de la Salud o de la *RPSP/PAJPH*.
